# Barriers to Providing Optimal Care in Idaho from the Perspective of Healthcare Providers: A Descriptive Analysis

**DOI:** 10.3390/healthcare13030345

**Published:** 2025-02-06

**Authors:** Alexis A. Ericsson, Allie D. McCurry, Lucas A. Tesnohlidek, B. Kelton Kearsley, Morgan L. Hansen-Oja, Gillian C. Glivar, Allie M. Ward, Kathryn J. Craig, Eva B. Chung, Skyler J. Smith, Tabarak O. Alomar, Luke A. La Mue, Karina S. Lopez, Jake R. Goodwin, Thinh T. Kieu, Audrey J. Dingel, Catherine M. Rockwell Hill, Madeline P. Casanova, Jonathan D. Moore, Ryan Wiet, Russell T. Baker

**Affiliations:** 1 WWAMI Medical Education Program, University of Idaho, 875 Perimeter Drive MS 4061, Moscow, ID 83844, USA; aerics@uw.edu (A.A.E.); addm1@uw.edu (A.D.M.); ltesno@uw.edu (L.A.T.); bkkears@uw.edu (B.K.K.); hansen64@uw.edu (M.L.H.-O.); gilliang@uw.edu (G.C.G.); award26@uw.edu (A.M.W.); craigka@uw.edu (K.J.C.); evachung@uw.edu (E.B.C.); smitsky4@uw.edu (S.J.S.); talomar@uw.edu (T.O.A.); llamue@uw.edu (L.A.L.M.); klopez7@uw.edu (K.S.L.); jgood1@uw.edu (J.R.G.); thinhq@uw.edu (T.T.K.); dingelau@uw.edu (A.J.D.); hillcm98@gmail.com (C.M.R.H.); mcasanova@uidaho.edu (M.P.C.); 2 School of Medicine, University of Washington, 1959 NE Pacific St., Seattle, WA 98195, USA; jonathandm@uidaho.edu; 3 Idaho Office of Underserved and Rural Medical Research, University of Idaho, 875 Perimeter Drive MS 4061, Moscow, ID 83844, USA; rwiet@uidaho.edu

**Keywords:** healthcare, interprofessional, rural

## Abstract

**Background/Objectives**: Few studies have assessed barriers to providing care from the perspective of interprofessional healthcare providers. Despite Idaho’s predominantly rural geography, limited research exists assessing barriers to providing care within the state. This study sought to assess barriers to providing optimal healthcare using a sample of 400 healthcare providers at 22 clinic sites across the state. **Methods**: A barriers to providing optimal care 9-factor, 41-item survey was modified from an existing survey. Healthcare providers rated barrier items using an 11-point Likert scale. The survey was distributed to a convenience sample of healthcare providers in 22 different clinic sites in rural Idaho. **Results**: Four hundred interprofessional healthcare providers in Idaho across 13 professional disciplines completed surveys. Items in the *Service Access* (mean = 7.14), *Patient Complexity* (mean = 6.59), and *Resource Limitations* (mean = 6.18) factors were reported as the most commonly perceived barriers to providing optimal care. **Conclusions**: Few studies have assessed rural interprofessional providers’ perceived barriers to providing optimal, high-quality, care, specifically in the rural state of Idaho, where healthcare services are often not equitable compared to urban regions. The results suggest that commonly perceived barriers exist throughout the state, particularly *Service Access*, *Patient Complexity*, and *Resource Limitations*. Further research is needed to develop data-driven decisions to address these concerns.

## 1. Introduction

Ensuring adequate access for the approximately 63 million individuals living in rural United States (U.S.) remains a pressing concern [1]. The U.S. Census Bureau defines “rural” as regions not classified as urban or metropolitan, a broad definition encompassing small towns with populations below 2500 and semi-urban zones with up to 49,999 residents [2]. These areas, which comprise 19% of the U.S. population and span 97% of the geographic region, are characterized by open countryside, geographical remoteness, and lower population densities [1]. Such characteristics contribute to the diverse nature of rural areas, posing challenges in navigating the healthcare system. Access to healthcare is a major determinant of health [3]. Optimal healthcare may be defined as delivering accessible, high-quality, patient-centered services that effectively address the physical, mental, and social needs of individuals while reducing barriers to care. For those living in rural areas, uncontrollable obstacles inherent to rural living can impede access to optimal healthcare services [4]. Challenges such as geographical isolation and topographical constraints impact disease patterns and overall wellness in rural areas [4].

Prior research has demonstrated that rural residents have poor access to care due to increased travel distance, lower likelihood to be motivated to travel significant distances, greater probability of experiencing financial burdens associated with travel, lower likelihood to have access to reliable transportation, and greater likelihood to miss appointments due to transportation issues [5]. Compared to their urban counterparts, rural populations face greater disparities, which are more pronounced in smaller rural areas than in larger ones [6]. For instance, rural individuals have a life expectancy of 76.8 years—two years less than urban individuals at 78.8 years [5]. Rural communities experience higher rates of health risk factors such as smoking, obesity, and inactivity [7]. Residents in these areas are also disproportionately affected by suicide, serious mental illness, and child and young adult mortality, stemming at least in part from a lack of access to mental healthcare and timely trauma care due to their rural location [8,9]. All these factors contribute to poorer health outcomes and elevated morbidity and mortality rates in rural areas as compared to urban areas [7].

Rural healthcare providers must deliver a broad range of healthcare services among a landscape of obstacles that affect their medical practice. Moreover, they are responsible for addressing the healthcare needs of populations disproportionately affected by risk factors and adverse health outcomes [7]. Additionally, this complex patient population is expanding. For example, between 2020 and 2021, nonmetropolitan areas experienced a growth rate surpassing urban areas for the first time since the mid-1990s [10]. Furthermore, net migration to rural communities remained positive through 2022, although at a slowed rate, with a proportionate decrease in urban growth, which may be associated with the increased prevalence of remote work opportunities following the COVID-19 pandemic [11]. There is also a noticeable trend of older adults retiring in rural areas while younger individuals migrate to metropolitan areas for work, contributing to the rapid aging of the rural population [7,11]. This demographic shift exacerbates the healthcare provider shortage in rural communities, where less than 10% of healthcare resources are available [12]. Primary care providers are challenged to provide optimal care due to declines in the primary care physician supply from 2005 to 2015 in rural counties, outpacing declines in urban counties by a loss of more than four physicians per 100,000 [13]. The shortage is even more pronounced for specialized care, with urban areas having between seven to eight times more practicing cardiologists, anesthesiologists, dermatologists, and gastroenterologists than rural communities [14]. The provider shortage leads to poor access to specialists, low follow-up adherence due to prolonged clinic wait times, and high patient turnover rates [5]. This widening gap in healthcare resources poses a significant challenge for rural healthcare providers striving to meet the growing needs of their communities.

Idaho, a predominantly rural state, faces distinct healthcare challenges. With a land area of 83,570 square miles and a population of 1,973,752, the state ranks 14th in size but 44th in population density, with approximately 22 people per square mile [15]. Nearly one-third of the state’s population lives in sparsely populated areas geographically isolated from urban population centers where most major healthcare centers reside [16]. Rural Idahoans face not only geographic barriers to accessing healthcare services but also demographic challenges, with the patient population being older, with higher poverty rates and uninsured rates compared to their urban counterparts [17]. The state’s severe healthcare provider shortage is another known concern. Idaho ranks 50th in the nation for the number of active and practicing physicians per capita [18]. The physician-to-patient ratio is unlikely to improve as Idaho is the fastest-growing state in the nation, with a population surge of 1.8% between 2021 and 2022 and quickly approaching a state population of 2,000,000 [19]. Furthermore, over one-fourth of Idaho physicians are over 60 and nearing retirement [20]. The geographical isolation of Idaho, paired with the shortage of healthcare resources, impacts the well-being of current and future Idahoans.

While most studies have focused on patients’ perspectives on barriers to receiving care, there is limited research exploring healthcare providers’ viewpoints. For example, a recent study conducted semi-structured interviews with 12 providers in Montana, while a larger study surveyed 1500 healthcare providers from Alaska and New Mexico [6,12], and one state-based study interviewed 20 rural primary care providers in Pennsylvania [21]. However, none of these studies included healthcare providers from Idaho. Despite these limitations, previous studies have made valuable contributions to rural healthcare research by identifying various barriers to delivering adequate care in rural areas. These findings highlight the importance of gathering insights from healthcare providers who are uniquely positioned to identify and address the challenges of practicing medicine in rural settings.

Other researchers investigating barriers to accessing optimal healthcare in rural America have conducted literature reviews focused on specific rural populations or medical services. One review described the barriers to access for the American Indian and Alaska Natives and highlighted the significant healthcare limitations for minority groups residing in rural America, although the exact states or regions included in the review were unclear [5]. Another review that included Idaho was specific to chronic pain management and demonstrated that rural patients were more likely to experience severe pain but were less likely to engage in pain management modalities such as physical therapy and psychological support [22]. Both literature reviews addressed specific, vulnerable rural populations that had challenges accessing optimal healthcare services rather than addressing the rural population in its entirety.

Further, few studies have used large samples of interprofessional healthcare providers to assess barriers to providing optimal care [23], and no such study focuses specifically on Idaho providers and the state’s unique rural health challenges (e.g., size, geographic constraints, population growth, etc.). In rural research, accurate region-specific data are crucial because barriers vary between communities due to factors including population size, geography, cultural composition, and economic conditions [24]. In a state as rural and under-resourced as Idaho, having up-to-date, region-specific data is essential to understanding and addressing healthcare accessibility challenges to support optimizing care to meet the healthcare needs of patients. The purpose of this study was to investigate the barriers to providing optimal care in Idaho from the healthcare providers’ perspective.

## 2. Materials and Methods

Identical paper and electronic surveys were created; electronic versions were distributed via Qualtrics (Qualtrics Inc., Provo, UT, USA), while paper responses were subsequently input into Qualtrics by the research team. The survey included a modified Barriers to Providing Optimal Healthcare in Idaho instrument and a participant demographic questionnaire. The cross-sectional study used a convenience sample of healthcare providers from 22 clinical sites in Idaho who participated between May and August of 2023. The surveyed clinical sites were primarily selected for participation as the sites were rural Idaho clinical sites hosting medical students participating in the University of Washington School of Medicine Rural Underserved Opportunities Program (RUOP); however, a few Idaho clinical sites not participating in the RUOP were selected to provide greater representation across Idaho. All healthcare providers at these sites who participated in the study practiced at rural facilities, while some may have practiced at these sites secondarily to their primary practices in urban communities. Clinical sites varied in size, composition of specialties, access to inpatient care providers, and type of healthcare organization (e.g., independent primary care clinics to multiple outpatient clinics and their associated hospitals that were part of a larger statewide healthcare organization). Surveys at all sites were distributed to healthcare providers in direct patient care roles via one of the following mechanisms: a distributed physical copy of the survey, a card/flyer with a QR code to the survey, an email with the survey link being to relevant staff members within the healthcare organization, or access via the inclusion of the survey link with a study description in the healthcare organization’s internal monthly newsletter. Participants provided informed consent before completing the survey and were compensated with a $25 Amazon gift card. The University of Idaho Institutional Review Board reviewed the project and certified the study as exempt.

### 2.1. Instrumentation

#### 2.1.1. Modified Barriers to Providing Optimal Healthcare in Idaho

The Barriers Experienced in Providing Healthcare instrument was generated from qualitative research, including interviews and focus groups, conducted in Alaska and New Mexico [6,25], but lacked psychometric testing to establish scale measurement properties [6]. For this study, the instrument was modified by the research team to eliminate two items based on the following considerations: items that did not match the factors proposed in the original survey [6], for brevity and clarity of phrasing, to reflect changes to accepted language for conditions (e.g., “addiction” was replaced with “substance use disorder”) [26], and to follow best survey practices (e.g., double-barreled items were split into two items) [27]. After modifications were made, the final instrument contained 41 items classified into 9 factors. The modified instrument retained the original Likert scale, and participants rated items using an 11-point Likert scale (1 = never a barrier, 11 = always a barrier) [6].

#### 2.1.2. Participant Demographic Questionnaire

Participants completed a 14-item demographic questionnaire. Items asked participants about sex, age, ethnicity, profession, specialty field, primary location of practice, secondary location of practice, status of current practice, specialty area, and years of experience.

### 2.2. Data Analysis

Data were exported from Qualtrics to the Statistical Package for Social Sciences Version 27 (SPSS, Inc., Chicago, IL, USA) for analysis. Responses missing more than 10% of the items in the survey (i.e., 4 items) were removed from the dataset. Individual responses with at least 90% of the data were retained, and individual missing items were replaced with the rounded mean score [28,29]. In line with the purposes of our study, categorical variables were assessed with frequencies and percentages, while a descriptive analysis was conducted to calculate the means and standard deviations for each proposed factor within the Modified Barriers to Providing Optimal Healthcare in Idaho survey.

## 3. Results

Four hundred individuals currently working as practicing healthcare providers in rural Idaho completed the survey. Most participants were nurses (*n* = 131, 32.4%) or physicians (*n* = 70, 17.3%), with the least represented of the 13 included professions being speech-language pathologists (*n* = 5, 1.2%). Respondents had an average of 13 years of clinical practice experience (range = 1–50), with a slight majority of participants having fewer than 10 years of clinical practice experience. Healthcare providers had primary practice locations in all seven Idaho public health districts, with most from Public Health District 2—North Central (*n* = 91, 22.5%) and Public Health District 6—Southeastern (n = 89, 22.0%). Additionally, respondents were aged 18–77 years (mean age = 41 ± 12), with females accounting for 74.0% (*n* = 299) and males accounting for 25.2% (*n* = 102) of the sample, with most (*n* = 319, 76.5%) reporting ‘White’ as their race or ethnicity. A full breakdown of participant demographics is presented in Table 1.

The mean scores and standard deviations of each of the seven factors in the scales are presented in Table 2. The most common factors cited as barriers perceived by healthcare providers in Idaho were *Service Access* (mean = 7.14), *Patient Complexity* (mean = 6.59), and *Resource Limitations* (mean = 6.18). Conversely, *Patient Avoidance* (mean = 4.45), *Outsider Role* (mean = 3.26), and *Confidentiality* (mean = 3.14) were perceived as the factors least likely to hinder the ability to provide care as barriers in rural Idaho (Table 2).

The means and standard deviations for group responses to each of the 41 items are presented in Table 3. Only the *Service Access* and *Patient Complexity* factors contained items where the mean scores were above 6 for all the items in the factors. Similarly, only the *Outside Role* and *Confidentiality Limitations* factors contained items where all the mean scores were below four for each of the items in the factor.

## 4. Discussion

Our study aimed to assess perceived barriers to delivering high-quality care to rural communities in Idaho. By surveying 400 interprofessional healthcare providers throughout the rural and underserved state, valuable information was gained providing the most comprehensive view to date of perceived barriers to providing care in Idaho. When the results were analyzed in combination with previous findings in Idaho and other rural areas, trends in the data emerged that helped illustrate the current status of healthcare in the state. The identification of the most common barriers to optimal care in Idaho can support future allocation of resources and initiatives to address the most pressing barriers and concerns of practicing healthcare providers to improve healthcare services deliver in Idaho.

### 4.1. Modified Barriers to Providing Optimal Healthcare Factor Breakdown

#### 4.1.1. Service Access Factor

The *Service Access* factor addressed concerns regarding patients’ ability to seek medical care due to geographical, transportation, or financial issues. Survey respondents indicated that *Service Access* was the most frequently experienced barrier, with a combined mean of 7.14. More specifically, providers rated the item “Distances that clients/patients needed to travel to receive specialty care”, the highest (mean 7.85). Past research supports this finding, as patients are less likely to travel for physician appointments if they need to travel over 30 min to receive care [30]. For more complex and specialized care, Idahoans living in urban areas have to travel 25 miles away to the nearest tertiary hospital [31]. In contrast, their rural counterparts must travel twice as far [31]. A study of Medicare beneficiaries in five states, including Idaho, found that median one-way travel for serious conditions (e.g., ischemic heart disease and cancer) was consistently greater than 30 min for small and isolated rural residents, exceeding one hour in 25% of cases [32]. The second most common item, “Inability of your clients/patients to afford treatment or care”, had a mean of 7.49. Regarding financial constraints, remote and rural counties in Idaho consistently have lower per capita incomes, higher unemployment rates and poverty levels, and smaller job growth, compared to more urban communities [31].

#### 4.1.2. Patient Complexity Factor

*Patient Complexity* explored patients’ understanding and appreciation of their mental and behavioral health and the influence this has on providers’ ability to provide optimal care. With a combined mean of 6.59, *Patient Complexity* was perceived as the second most frequent barrier. This factor also had the largest standard deviation (*n* = 2.51), suggesting that rural providers may have varying opinions or perspectives on *Patient Complexity.* One explanation for the sizeable spread in responses is that patients may not adequately address their mental health concerns with providers as rural residents often stigmatize mental health and are skeptical of related medical interventions [23]. It is also possible that, as residents of rural areas themselves, providers may be subject to the same stigmatizing attitudes toward mental health prevalent in their communities and may not appropriately address mental health concerns with their patients. Additionally, the federal government has classified the entire state of Idaho as a Mental Health Professional Shortage Area [18]. Rural areas tend to have significantly fewer psychiatrists and psychiatric nurse practitioners than urban areas, leaving providers who are not specialized in mental and behavioral health with the responsibility to care for patients with these conditions on a temporary or permanent basis [8,33]. The combination of social stigma and overall lack of access to mental health services likely contributes to healthcare providers’ concern for their patients’ mental well-being.

#### 4.1.3. Resource Limitations Factor

Items related to adequate access to expert providers and proper facilities were included in the *Resource Limitations* factor. With a combined mean of 6.18, *Resource Limitations* was rated as the third most likely factor to be perceived as a barrier. Throughout the U.S., urban counties tend to have greater concentrations of physicians than rural counties [34]. In 2010, 12% of hospitalizations, 11% of days of care, and 6% of inpatient procedures were provided in rural hospitals despite almost one-fifth of Americans living in these rural areas [23]. Specifically, within Idaho, 97.7% of Idaho’s counties are designated primary care shortage areas and five counties still do not have any practicing physicians [18,33]. Thus, there are fewer physicians providing direct patient care to rural areas compared to urban areas with the greatest disparities existing in isolated small rural communities [33]. One specialty with a profound discrepancy is OBGYN where 85% of physicians are concentrated in the state’s seven most populated counties [35]. The net supply of practicing OBGYNs decreased from 268 in August of 2022 to approximately 210 in November of 2023 and currently only 22 of Idaho’s 44 counties have active OBGYNs [35]. The remaining 210 physicians are providing care to nearly one million women in the state [35].

#### 4.1.4. Training Constraints Factor

The *Training Constraints* factor evaluated the staff’s ability to access further training and had a combined mean of 4.98. Of the six items assessed, “Staff members who were not sufficiently trained or experienced” was perceived most commonly as a barrier with a mean of 5.49. Overall, this finding is consistent with previous research where it was indicated that the smaller the community, the greater the burden of receiving adequate training [6]. Additionally, more rural primary care providers reported having personal knowledge of an impaired or incompetent physician than their urban peers [36]. Prior research has identified the largest contributors to the lack of rural training to be geographic isolation, lack of funding, and no paid time off [37]. Efforts are being made within Idaho to provide rural healthcare providers with continuous learning opportunities. For example, ECHO Idaho is a free tele-education and tele-mentoring educational program where rural healthcare providers are connected with specialists across the state to increase their knowledge and clinical competence to treat complex diseases [38].

#### 4.1.5. Time Constraints Factor

The *Time Constraints* factor assessed issues related to having insufficient time to best care for patients and participant responses produced a combined mean of 4.92. Of the six items assessed, “Excessive practice demands on you that reduced your availability to provide care for clients/patients” was perceived most commonly as a barrier to healthcare, with a mean of 5.40. These demands impact the quality of care as increased time pressure is associated with a lower number of diagnoses recorded in a visit, more planned and unplanned follow-up visits, and an increased likelihood of subsequent hospitalization [39]. Healthcare providers endorsing excessive practice demands is not surprising, given Idaho’s scarce medical resources. Idaho has the lowest density of physicians providing direct patient care at 174 physicians per 100,000 residents, compared to the national average of 248 physicians per 100,000 residents [33]. Unfortunately, this shortage may only become magnified in the future. Idaho’s mean age of practicing physicians is 52 years old, meaning many physicians are nearing retirement age [33]. Additionally, Idaho’s healthcare demands are rapidly increasing due to being among the fastest-growing states in the nation. If there is not a strong rise in the number of healthcare providers in Idaho, time constraints will continue to reduce their availability to best care for patients.

#### 4.1.6. Overlapping Roles Factor

Challenges healthcare providers face due to living in the rural communities wherein they practice were included in the Overlapping Roles factor with participants’ responses resulting in a combined mean of 4.64. Of the three items assessed, the top two were “Client/patient desires to use non-standard or alternative treatment approaches”, and “Clients/patients seeking advice or help from you when you were in the community in non-professional settings”. Previous research has indicated that rural patients use alternative medicines more than their urban counterparts [40]. Caring for patients receiving alternative therapies can be challenging because the provider must consider medication interactions, treatment interference, and difficulty in monitoring progress, while still maintaining a trusting relationship with the patient. Healthcare providers also often live in the community in which they practice. Living in small communities with your patients makes unplanned client/patient and healthcare provider interactions inevitable. Sometimes these interactions result in patients wanting to discuss their current healthcare needs, making it challenging for healthcare providers to maintain appropriate professional boundaries [41]. Rural physicians were also more likely to report providing care for patients with whom they have a financial relationship and asked patients for monetary donations at higher rates than those practicing in urban areas [37]. These dual relationships complicate the patient–provider relationship for both the healthcare providers and their patients, which can be a barrier to providing optimal care.

#### 4.1.7. Client/Patient Avoidance of Care Factor

The combined mean for *Client/Patient Avoidance of Care* items was 4.45. Of the seven items in the *Client/Patient Avoidance of Care* factor, “Clients/patients lack of support from family or friends” was most perceived to be a barrier to providing care, with a mean of 5.63. In a previous survey of households in rural counties in North Carolina, respondents who received transportation from family or friends received 1.58 more visits for chronic care [42]. Due to the severely limited or often complete lack of public transportation options in rural Idaho, most patients requiring healthcare have to travel via private vehicle [31]. For patients unable to obtain appointments independently, namely the elderly and individuals with low incomes, family and friends can provide a critical link to care [31]. This is particularly true for patients requiring treatment from tertiary care facilities, as private insurance typically does not pay for caregiver travel expenses [31]. Limited patient access to social support can interfere with their ability to reach healthcare facilities, creating a serious barrier to providing care. Residents of rural communities, in general, report avoiding care at 1.7 times higher rates compared to those in urban areas, particularly in patients who are male, in younger age groups, or are uninsured [43]. As our study focused on the perspective of healthcare providers, it may not fully capture the extent of this barrier, as patients who are avoiding care may not be consistently interacting with the health system.

#### 4.1.8. Outsider Role Factor

The *Outsider Role* factor aimed to ascertain the extent to which healthcare providers viewing themselves as outsiders within the community was perceived as a barrier to providing care. The combined mean was 3.26, which was the second lowest of the nine factors. The item within the *Outsider Role* factor with the highest mean was “Inability to consult community leaders to increase your ability to provide care for your clients/patients” (mean = 3.69). A possible explanation for this being the second-lowest perceived barrier to providing care is that healthcare providers may be unaware of patients who are avoiding seeking care due to a perceived lack of cultural competency. For example, the LGBTQ+ community is a population with unique health needs whose care can be significantly impacted by culturally sensitive care, or lack thereof. Researchers examining healthcare for rural LGBTQ+ individuals reported the lack of cultural competency in rural providers may limit options for accessing care in rural communities [44]. Furthermore, in a survey of LGBTQ+ identifying youth in Idaho in 2022, it was reported that 55% of respondents wanted mental healthcare in the past year but were not able to obtain it, with those not receiving care often citing being afraid to discuss their mental health concerns with someone else (48%) and being afraid they wouldn’t be taken seriously (37%) [45]. Thus, patients who perceive a lack of cultural competency among healthcare providers may be less likely to seek care, which may result in the *Outside Role* factor being less perceived by providers as a barrier. A perceived lack of cultural competency among providers may result from insufficient training in relevant cultural competency areas among healthcare professionals. For example, the median total instruction time across all four years of medical school regarding LGBTQ+ health was recently reported to be a total of 11 h [46], while a 2011 study found one-third of medical schools in the U.S. and Canada provided zero hours of curriculum devoted to this topic [47]. Given the age demographics of Idaho primary care providers, it is possible that little to no training was provided during their health professions training on some of the relevant cultural competency challenges that patients currently face [33,46,47].

However, a positive potential explanation for participants who reported the *Outsider Role* less frequently as a barrier to providing care may be related to the demographics and experience of healthcare providers in Idaho. Of the thirteen healthcare professional disciplines surveyed, nurses had the highest number of participants. Statewide, over one-quarter of Registered Nurses (RNs) are age 55 or greater [48]. Additionally, 42.8% of sample interprofessional providers reported practicing for eleven years or more. Therefore, as Idaho clinicians have a wealth of professional experience and potential time within a community, it is possible that those individuals form more ties to the community, including connections to important local leaders, reducing their perception of being an outsider [49].

#### 4.1.9. Confidentiality Limitations Factor

The factor least perceived to be a barrier to providing care was *Confidentiality Limitations*, with a combined mean of 3.14. Of the four items within the factor, the item with the highest mean was “limited ability to assure confidentiality in the local community” (mean = 3.49). In rural communities, it is nearly impossible for interactions with patients to be limited to the clinical setting [49]. Rather, patients and providers routinely encounter each other in informal settings as friends, neighbors, parishioners at church, or patrons at a local restaurant [49]. While it is difficult for healthcare providers to navigate these dual relationships while maintaining boundaries, it can also prevent patients from seeking care due to hesitancy to share sensitive medical concerns with people they know personally [23,49]. Additionally, past experiences with breaches of confidentiality can cause longstanding distrust toward the medical community that is difficult for subsequent providers to rebuild [49]. These incidents may be more common in rural areas as a national survey of physicians regarding medical professionalism found that rural physicians were more likely than urban physicians to report accidentally or purposefully revealing personal health information about a patient to an unauthorized person [36]. *Confidentiality Limitations* may also have been viewed as less of a limitation as some patients may seek the option to travel further from their hometown to access healthcare in a different town, perceiving this as a way to ensure higher confidentiality; however, for many, confidentiality is viewed as more of a luxury compared to other essential healthcare traits [6].

### 4.2. Limitations and Future Studies

Although our study included a large sample of Idaho healthcare providers, it has limitations, particularly when using a convenience sampling method. First, while all sites were in rural locations, most sites were connected to an academic teaching institution, potentially over-representing settings with a stronger focus on education, while some sites included providers who practiced in both rural and urban locations within a health system. Second, the sample was disproportionately composed of primary care providers, which may limit the generalizability of findings to other specialties. Third, response sample sizes varied by site, which may have resulted in more responses from certain clinics or regions within Idaho, influencing the sample’s representativeness of barriers across different regions of Idaho. These potential limitations should be considered when interpreting the results and generalizing findings beyond the specific study. Additionally, as participants were required to practice in rural areas, responses from providers who exclusively practiced in urban areas in Idaho were excluded, decreasing the generalizations that can be applied to the urban areas of the state. Thus, conclusions cannot be extrapolated to the entire state as large metropolitan areas, where 70% of urban dwellers and many of the rural residents seek primary and specialized care, were not included in this study, and narrows some of the generalizability of the findings to only rural healthcare settings [16].

Also, most surveyed sites had inadequate contact with health specialists, including but not limited to, behavioral health providers and surgical specialists, because many of the sites did not offer these services, and access to these providers for study participation was more difficult. Thus, the perceptions reported may not fully represent the perceptions of specialty providers and may underrepresent barriers to accessing care (e.g., mental health and specialty services are lacking in these areas [5,14]). Furthermore, the medical setting varied by clinical site and types of providers present. For example, some sites included both inpatient and outpatient care, while other sites only included outpatient care. While we believe this best represents the current state of healthcare in Idaho, the added variability to the data may need to be explored further. Another limitation was an uneven distribution of responses across public health districts. While we believe this aligns well with the state demographics, the results may impact the generalizability of these findings as different parts of the state vary in distance from access to major medical centers, and barriers may vary based on geographic location.

The demographic responses collected also raise concerns about the comprehensiveness of the perspectives captured (e.g., only 70 physicians). However, the demographic breakdown of the survey mirrors the current state of practice and, therefore, likely did not greatly bias our results. For example, women make up 75% of total hospital employment, so it was unsurprising that 74% of our sample respondents were females [50]. Additionally, of the respondents, nurses constituted the largest group within our sample at 32.4%, aligning with their status as the largest sector in hospital employment [51]. Lastly, it is important to acknowledge that while this study explored barriers to providing care, it did not delve into barriers perceived by the patient, potentially affecting our knowledge and understanding of the healthcare challenges that plague these communities.

Subsequent studies should address these limitations to provide a more comprehensive understanding of rural healthcare challenges in Idaho. Additionally, research should investigate rural areas with more positive health outcomes to identify their strengths, along with identifying the gaps in communities that lack certain healthcare needs. Continued research efforts should assess concerns faced in these underserved communities as well as establish evidence that can be used to implement solutions to overcome these barriers. Further research could also include Idaho’s larger metropolitan areas to determine the barriers to providing optimal care in those communities and compare them to the findings in rural areas to inform statewide initiatives and policies.

## 5. Conclusions

The purpose of our study was to assess the barriers to delivering optimal healthcare within the state of Idaho. The top three perceived barriers to providing optimal care as perceived by rural primary care providers in rural Idaho were *Service Access*, *Patient Complexity*, and *Resource Limitations*. Future research should continue to collect additional information related to barriers to providing optimal care so data-driven decisions can be made that will improve healthcare for Idahoans, particularly in rural and under-resourced regions. Particularly, future research examining how barriers to Service Access and Resource Limitations can be improved through various strategies, such as increased use of telehealth and remote consultation of specialists, novel transportation strategies for rural patients, broader distribution of ECHO Idaho materials for tele-education, and further investment in programs to train healthcare professionals from rural areas and incentives for providers to promote practicing in rural communities, would be valuable.

## Figures and Tables

**Table 1:** Breakdown of participants demographics.

Characteristics	Number of Participants	Percentage of Participants (%)
Profession		
Nurse (e.g., RN)	131	32.4
Physician (i.e., MD/DO)	70	17.3
Medical assistant	66	16.3
Physician assistant (i.e., PA)	25	6.2
Nurse practitioner (i.e., NP)	21	5.2
Certified nurse assistant	19	4.7
Physical therapist	13	3.2
Social worker (e.g., LCSW)	9	2.2
Pharmacist (i.e., PharmD)	8	2
Respiratory therapist	6	1.5
Occupational therapist	6	1.5
Speech language pathologist	5	1.2
Idaho Public Health District		
District 1—Panhandle	51	12.6
District 2—North Central	91	22.5
District 3—Southwest	44	10.9
District 4—Central	65	16.1
District 5—South Central	38	9.4
District 6—Southeastern	89	22.0
District 7—Eastern	26	6.4
Years of clinical practice		
≤10 years	174	43.5
≥11 years	171	42.8
Primary practice populations grouped		
Small ≤ 4999 people	154	43.5
Large ≥ 5000 people	246	42.8
Sex		
Female	295	73.8
Male	102	25.5
Prefer not to answer	3	0.8
Race or ethnicity		
White	319	76.5
Hispanic or Latino or Spanish origin	35	8.4
American Indian or Alaska Native	32	7.7
Black or African American	5	1.2
Asian	4	1.0
Native Hawaiian or Other Pacific Islander	3	0.7
Other	7	1.7
Prefer not to answer	12	2.9

**Table 2:** Means and standard deviations for each surveyed factor.

**Factor**	**Mean**	**Standard Deviation**
Service Access	7.14	2.34
Patient Complexity	6.59	2.51
Resource Limitations	6.18	2.37
Training Constraints	4.98	2.38
Time Constraints	4.92	2.37
Overlapping Roles	4.64	2.46
Client/Patient Avoidance of Care	4.45	1.94
Outsider Role	3.26	1.91
Confidentiality Limitations	3.14	1.97

**Table 3:** Means and standard deviations for each surveyed item.

Factor	Item	Mean	Standard Deviation
Service Access	Distances that clients/patients needed to travel to receive specialty care	7.85	2.60
	Inability of your clients/patients to afford treatment or care	7.49	2.68
	Lack of access to transportation by clients/patients who needed to travel to receive healthcare	6.80	2.72
	Distances that clients/patients needed to travel to receive general care	6.48	2.70
Patient Complexity	Mental illness that co-existed with another serious problem/illness	6.78	2.70
	Clients/patients’ difficulty in understanding the links between their behavior and their needed care	6.76	2.62
	Substance use disorder that co-existed with another serious problem/illness	6.25	2.64
Resource Limitations	Limited access to consultants for specialty areas	6.84	2.93
	Limited number of providers within the community	6.62	2.81
	Lack of appropriate treatment facilities in the community	6.56	2.82
	Limited provider expertise in the community for certain problems/illnesses	5.78	2.96
	Limited access to consultants for ethical issues	5.18	3.19
Training Constraints	Staff members who were not sufficiently trained or experienced	5.49	2.99
	Limited time/coverage to have breaks during the workday	5.47	3.37
	Limited time/coverage to allow you to receive additional training	5.31	3.14
	Limited time/coverage to have vacation/paid time off	4.92	3.09
	Limited access to training to increase your skills and abilities	4.62	2.83
	Limited access to training to keep yourself up to date	4.12	2.76
Time Constraints	Excessive practice demands on you that reduced your availability to provide care for clients/patients	5.40	2.95
	Insufficient time to fully learn about client/patient	5.03	2.76
	Insufficient time to fully learn about client/patient values or preferences pertaining to their care	4.90	2.82
	Insufficient time to fully discuss or explain to clients/patients about their problem/illness or treatment	4.90	2.87
	Having to work at a pace that was not comfortable for your clients/patients	4.75	2.51
	Having to work at a pace that was not comfortable for you	4.64	2.56
Overlapping Roles	Client/patient desires to use non-standard or alternative treatment approaches	4.92	2.71
	Clients/patients seeking advice or help from you when you were in the community in non-professional settings	4.82	2.99
	Having to interact in non-professional roles in the local community with your clients/patients present	4.23	2.87
Client/Patient Avoidance of Care	Clients/patients lack of support from family or friends	5.63	2.53
	Stigma or embarrassment that clients/patients experienced in the community if people had known of their problem/illness	5.10	2.72
	Clients/patients who avoided providing or withheld necessary information	4.58	2.39
	Clients/patients who became defensive or avoided or prematurely terminated care	4.38	2.42
	Client/patient fear of partner/spouse or family response if help was sought	4.08	2.33
	Clients/patients who were concerned that seeking treatment might get them in legal trouble or trouble with social services	3.76	2.44
	Clients/patients who were concerned that providing necessary information for treatment might get them in legal trouble or trouble with social services	3.67	2.43
Outsider Role	Inability to consult community leaders to increase your ability to provide care for your clients/patients	3.69	2.59
	Inability to learn more about local values and customs	3.08	2.09
	Your hesitance or discomfort with talking about stigmatizing problems with your clients/patients	3.03	2.13
Confidentiality Limitations	Limited ability to assure confidentiality in the local community	3.49	2.52
	Clients/patients concerns of staff members breaching their confidentiality	3.25	2.42
	Staff or colleagues having access to sensitive records about your clients/patients	3.08	2.39
	Legal or ethical requirements to report certain information about your clients/patients to outside authorities	2.76	1.99

## Data Availability

The datasets analyzed during the study are not publicly available per study protocol; however, deidentified data may be available from the corresponding author with permission from the University of Idaho upon reasonable request.
